# Hierarchically Structured Surfaces Prepared by Phase Separation: Tissue Mimicking Culture Substrate

**DOI:** 10.3390/ijms23052541

**Published:** 2022-02-25

**Authors:** Markéta Kadlečková, Kateřina Skopalová, Barbora Ptošková, Erik Wrzecionko, Eliška Daďová, Karolína Kocourková, Aleš Mráček, Lenka Musilová, Petr Smolka, Petr Humpolíček, Antonín Minařík

**Affiliations:** 1 Faculty of Technology, Tomas Bata University in Zlin, 760 01 Zlin, Czech Republic; m1_kadleckova@utb.cz (M.K.); b_ptoskova@utb.cz (B.P.); wrzecionko@utb.cz (E.W.); kkocourkova@utb.cz (K.K.); mracek@utb.cz (A.M.); lmusilova@utb.cz (L.M.); smolka@utb.cz (P.S.); 2 Centre of Polymer Systems, Tomas Bata University in Zlin, 760 01 Zlin, Czech Republic; skopalova@utb.cz (K.S.); e_dadova@utb.cz (E.D.)

**Keywords:** phase separations, phase inversion, surfaces, hierarchically structured, foams, line-specific response, stem cells

## Abstract

The pseudo 3D hierarchical structure mimicking in vivo microenvironment was prepared by phase separation on tissue culture plastic. For surface treatment, time-sequenced dosing of the solvent mixture with various concentrations of polymer component was used. The experiments showed that hierarchically structured surfaces with macro, meso and micro pores can be prepared with multi-step phase separation processes. Changes in polystyrene surface topography were characterized by atomic force microscopy, scanning electron microscopy and contact profilometry. The cell proliferation and changes in cell morphology were tested on the prepared structured surfaces. Four types of cell lines were used for the determination of impact of the 3D architecture on the cell behavior, namely the mouse embryonic fibroblast, human lung carcinoma, primary human keratinocyte and mouse embryonic stem cells. The increase of proliferation of embryonic stem cells and mouse fibroblasts was the most remarkable. Moreover, the embryonic stem cells express different morphology when cultured on the structured surface. The acquired findings expand the current state of knowledge in the field of cell behavior on structured surfaces and bring new technological procedures leading to their preparation without the use of problematic temporary templates or additives.

## 1. Introduction

Surface treatment is one of the most important post-processing techniques in many industries. In terms of changes in the chemical composition of the surface, these treatments can be divided into two groups. The first includes the deposition of cover layers with a different chemical composition compared with the treated material. The second group includes modifications based on changes to the structure of the original material surface.

Surfaces that are structured at multiple levels are called hierarchically organized surfaces [1,2,3]. A typical example of such surfaces in nature is the lotus leaf [4,5], which is characterized by self-cleaning properties and superhydrophobic character [6,7,8]. Hierarchically structured surfaces and porous structures are commonly used in biochemistry, electronics and optics [9,10,11,12]. They can serve as micro-reactors [13], scaffolds [14,15] and cell capture sites [10,16,17,18]. They affect cell adhesion, differentiation, proliferation and gene expression [9,19,20,21].

The effect of materials on cellular behavior depends on the scale of its structure. In general, it can be stated that the microstructures mainly affects the morphology and cytoskeleton of cells, whereas nanostructured surfaces often play a role in influencing cells differentiation and proliferation [22]. The study of Papenburg et al. investigated the effect of micropatterned substrates on mouse fibroblast proliferation. It was found that the orientation and growth of the cells was influenced by the design of the surface microstructure, i.e. the cells grew in the direction of the microfibers [23]. Gerecht et al. used nanostructured surfaces to affect the proliferation of human embryonic stem cells (hESC). hESCs grown on nanostructured poly (dimethyl siloxane) surface proliferated with increased alignment and elongation compared to flat substrate [24]. However, the response of cells to structured surfaces strongly depends on the type of cells used. For example, Leclerc et al. cultured two different cell lines on micro-grooved substrates. Mouse fibroblasts favored the surface of the groove ridge, whereas epithelial cells preferred space in the grooves. Due to this, there is also the possibility of using structured materials for co-cultivation of cells [25]. Here, 4 different cell lines (mouse embryonic fibroblast, human lung carcinoma cells, human epidermal keratinocytes and mouse embryonic stem cells) were tested on hierarchically structured surfaces to determine the specific cellular response.

For the preparation of defined surface reliefs, templates of various types of forms [26,27], clusters of colloidal particles, polymer mixtures [28,29] and photopolymerized layers [30] can be used. The disadvantage of these approaches is the need to remove the template afterwards and the risk of surface contamination. A possible solution to this problem is the use of molding instrument that can be converted to the gas phase after the required relief has been fixed. In practice, gaseous foaming agents [31], freezing approaches [32] and phase separation processes [2,33,34] are commonly used.

The literature describes a wide range of phase separation methods on polymer surfaces leading to the formation of different types of surface reliefs and pores [2,35,36]. Phase separation can be observed in both dry and wet casting [37] processes. From a physicochemical point of view, phase separation can be caused by shear forces [38], temperature changes [39], chemical reactions [40] and poor solvents [18,41,42]. 

This work deals with the problem of phase separation processes caused by the action of a poor solvent [2,34,43]. In this case, the drops of poor solvent act as template for the resulting relief. Regarding the method of deposition of good and poor solvent, there are two main modification approaches. The first is based on the condensation of poor solvent vapors on a pre-swollen polymer surface [2,34]. The second is associated with applying a mixture of good and poor solvent to a solid polymer surface [18,33,44].

Within this work, a previously published approach based on time-sequenced dosing of a solution mixture on a rotating polymer surface [33] is further developed. New approaches leading to the formation of hierarchically structured surfaces without the need of using additives and other problematic removable molding agents are discussed. The method of preparing foams is demonstrated on the example of a mixture of good and bad solvent with polymer content. It is shown that thickness of this foam can be controlled by the number of deposited doses of solution on rotating surface. A relatively simple, rapid and reproducible method of preparation of surface suitable for cell tests is introduced. The acquired findings expand the current state of knowledge in the field of the influence of material surfaces on cells behavior [10,45,46].

## 2. Results and Discussion

### 2.1. Hierarchically Structured Surfaces

**Figure 1:**
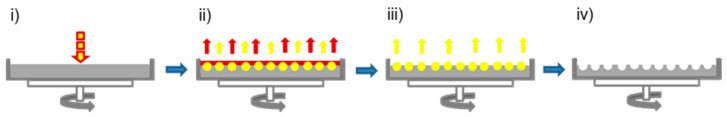
Principle of polymer surface texturization using phase separation associated with different evaporation rates of good and bad solvents at the rotation. (**i**) dosing of tetrahydrofuran (THF) with 2-ethoxyethanol (ETH) or water (H_2_O) on the polystyrene (PS) Petri dish; (**ii**) swelling and dissolving of polymer surface by good solvent (THF) and separation of poor solvent (ETH or H_2_O) into microdroplets; (**iii**) rapid evaporation of THF while the ETH or H_2_O droplets are embossed into the surface creating a template for the resulting structure; (**iv**) resulting structured PS surface.

The preparation of porous and hierarchically structured surfaces systematically corresponds to the procedure discussed in our previous work [33]. In contrast to the phase separation approaches described in the literature [2,18,34,47], this procedure is unique especially by the gradual, time-sequenced dosing of a multicomponent solvent mixture onto a rotating substrate. The principle of this process can be illustrated in a simplified manner in Figure 1.

In this work, polystyrene (PS) surfaces were treated with a mixture of tetrahydrofuran (THF) with 2-ethoxyethanol (ETH) or water (H_2_O) (Figure 1i). A good solvent (THF) swells and dissolves the polymer surface, while a poor solvent (ETH or H_2_O) separates into microdroplets (Figure 1ii). Subsequently, the THF evaporates rapidly (Figure 1iii), while the ETH or H_2_O droplets embossed into the surface create a “template” for the resulting structure (Figure 1iv).

**Figure 2:**
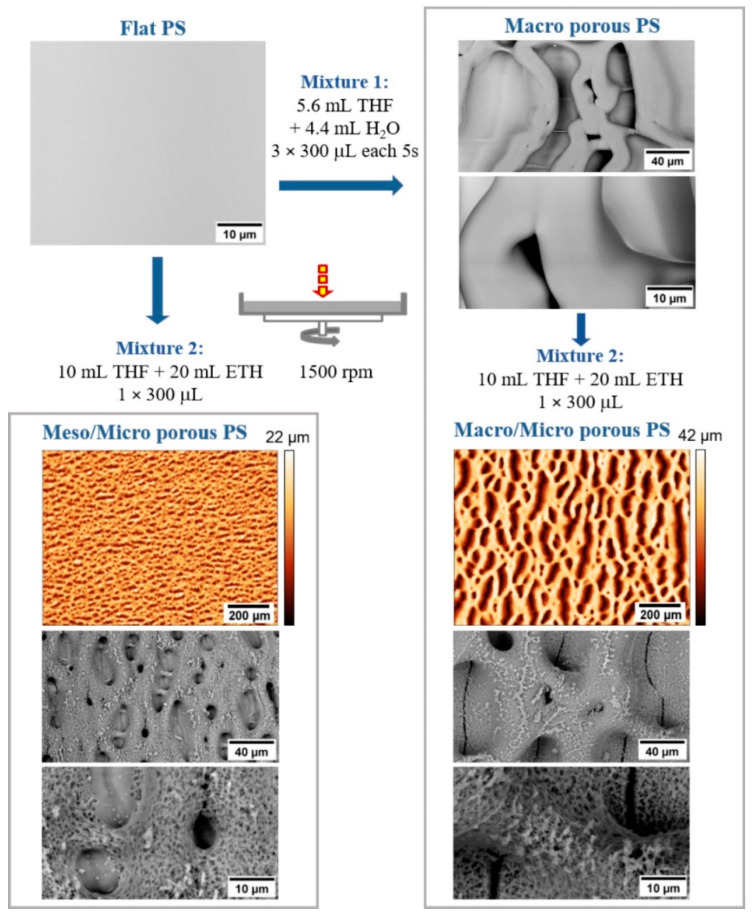
Hierarchically structured PS surfaces prepared by one- or two-step deposition of mixed solutions. Images from SEM and optical profilometer.

In summary, this process can be described as follows: diffusion of THF into PS; growth of the surface-swollen layer of PS; phase separation of ETH versus PS with THF; embossing ETH microdroplets into the PS surface with THF; reforming (aggregation) of ETH microdroplets; deformation of the shape of the ETH microdroplets due to rotation; gradual evaporation of THF and ETH from the surface; surface relief fixation [33]. Deformation of originally circular micropores on the PS surface, Figure 2, is associated with the coalescence of microdroplets of poor solvent into large units [2], sample rotation, rapid gas flow [48], and the viscoelasticity of systems [28].

From the findings presented in the previous work [33] and some others [2,18] it follows that the type of emerging surface texture is determined by temperature, speed of rotation, ratio and type of components in the mixed solution. Another possibility how to extend the type spectrum of emerging surface structures is a multi-step modification combining different types of mixed solutions, Figure 2. With such an approach it is possible to prepare hierarchically textured surfaces based purely on PS. This means that there is no need to add other additives to the polymer system as described in the literature [2]. Moreover, preparation procedures of hierarchical PS substrates surfaces were highly reproducible and the prepared surfaces were homogenously covered on 80 % of area with Micro, Meso/Micro and Macro/Micro pores. Only the peripheral area, close to the edges, was excluded from the analysis because of the accumulation of solvents and the presence of artefacts resulting from residual stresses in the substrate material [33]. All samples were, due to cell tests, prepared at least 40 times. 

Meso/Micro and Macro/Micro surfaces: To prepare the hierarchically structured surface shown in Figure 2, a macroporous surface was formed in the first step using a mixture of THF and H_2_O, similar to the work of E. DeRosa [18]. In the second step, a single dose of a mixture of THF and ETH was applied to the surface. Deposition of the second type of solvent mixture results in the formation of a specific microtexture on the primary macropores surface, as in the bottom right of Figure 2. The secondary microstructure can only be obtained by deposition of one dose of Mixture 2 during rotation. Repeated deposition (2 or more doses) of THF with ETH leads to dissolution of the primary macropores. For comparison, Mixture 2 was used directly to modify the flat PS surface. After the deposition of one dose on the rotating surface, a specific corrugation is formed, characterized by medium sized (Meso) pores with a size of 10 to 50 µm separated by wide interfaces with depressions of 0.1 to 10 µm, as in the bottom left of Figure 2. Repeated deposition of Mixture 2 on a flat rotating surface results in the sintering of medium-sized pores formed in the first step, like the Macro porous surface. For more information about pore size distribution and roughness analysis, see the supplement information.

**Figure 3:**
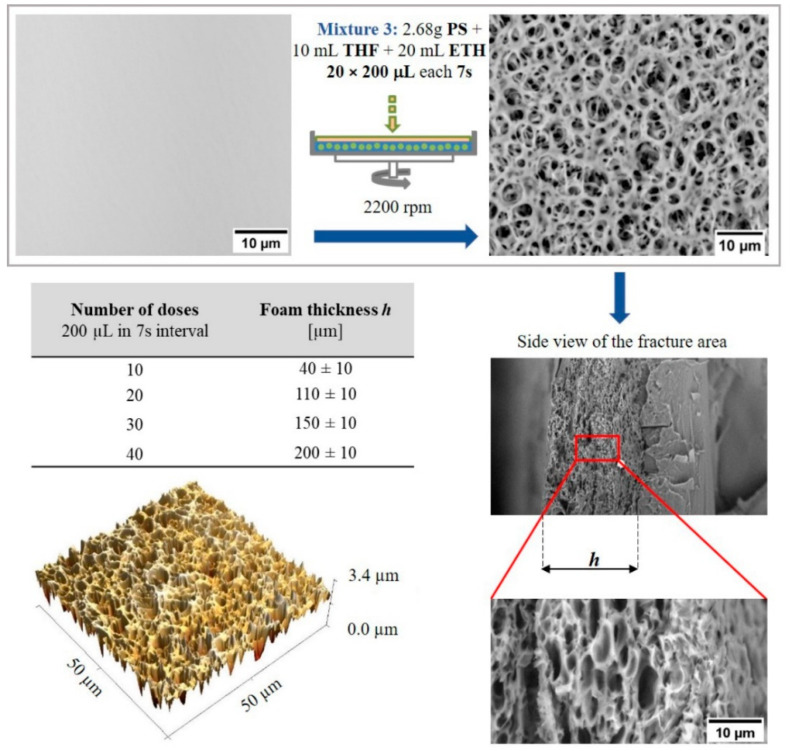
Porous PS layer prepared by time-sequenced phase separation (inversion) from a solvent mixture containing PS. 3D image obtained using AFM. Other images from SEM.

Foam like (Micro) surfaces: Time-sequenced phase separation under rotation [33] can be applied to prepare foam like (Micro) structures. For this purpose, it is necessary to add a polymer (PS) to the modification mixture. Added PS precipitates during the rapid evaporation of THF and separates around the ETH droplets. From a physicochemical point of view, this approach corresponds to the phase inversion described in the literature [36,49,50]. A fundamental challenge in this approach is to find a suitable ratio of the components of the mixture THF + ETH + PS (Mixture 3), so that the amount of ETH is just below the limit of the beginning of the separation of PS from the mixture and the concentration of PS in THF is sufficient to form a porous layer. The low concentration of PS does not allow the formation of sufficiently thick walls separating the individual pores in gradually applied layers. On the contrary, the high concentration of PS does not allow the addition of a sufficient amount of ETH. The procedure for preparing such a mixture is based on the gradual addition of ETH to a solution of PS in THF up to the limit of PS separations. This means that the ratio of components in Mixture 3 must be set up so that, once deposited on the surface, the individual components of the system are separated as quickly as possible. Multiple time-sequential dosing of the mixed solution overlaps the individual layers and increases the total thickness h of the foam like layer, as shown in the table in Figure 3. For more information about pore size distribution and roughness analysis, see the supplement.

In order to avoid sintering of the formed porous layers, it is necessary to correctly set the surface rotation speed, the volume of the dosed mixture and especially the time delay between doses of Mixture 3. A short time delay results in a cracked surface with minimum pores due to insufficient time for formation and fixing PS walls between ETH drops. On the contrary, a long-time delay results in the overlap of the formed pores due to the evaporation of the ETH before the deposition of the next layer. For a selected mixture of 2.68 g PS in 10 mL THF with 20 mL ETH, a dosing time interval of 5 s to 10 s can be used.

### 2.2. Influence of Structured Surfaces on Cell Behavior

In previous work [33], it was found that specifically microtextured surfaces created by using a mixture of THF and ETH can better simulate the native environment for adherent cell types (NIH/3T3). In addition to the surface texture, in the case of PS it is necessary to consider the wettability of the surface with water. This parameter was controlled both for the starting surfaces and for the plasma treatments and depositions of the culture medium. The results in Table 1 show that the structured PS surfaces exhibited comparable wetting characteristics with water at the time of cell deposition.

**Figure 4:**
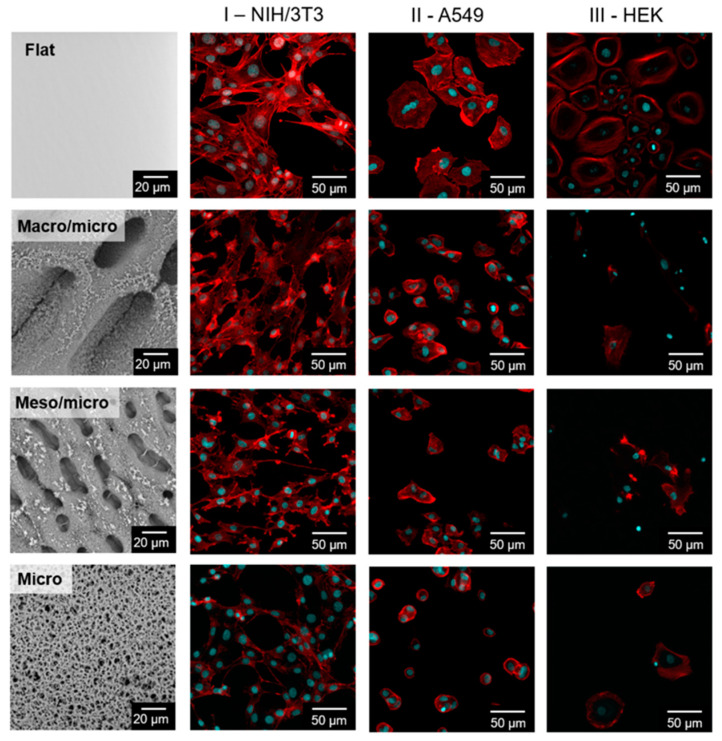
Different cell lines (NIH/3T3 (fibroblasts) column I, A549 (human carcinoma cells) column II and HEK (keratinocytes) column III) on the structured PS. Blue marked cell nuclei (Hoechst), red cytoskeleton (ActinRed). PS surfaces prepared by one or more stepwise time-sequenced phase separation (Macro/Micro and Meso/Micro) correspond to Figure 2. PS foam (Micro) prepared by phase inversion corresponds to Figure 3. SEM images (left), confocal microscope images in the columns I, II, III.

The behavior of cells is not only conditioned by the presence of pores with an average size in the order of tens of micrometers. An important role is played by the secondary microstructure at the interface of the individual meso and macro irregularities. Four different cell lines were used to determine the line-specific response of cells to hierarchically structured surfaces. As observed in Figure 4 HEK on individual surfaces were not able to proliferate well (Figure 4 column III). The low ability to proliferate in this case was probably related to the size of the pores, which range from 5 to 150 µm. For keratinocyte culture, the pores should not exceed a few micrometers in order to limit cell migration into the pores [51].

The A549 cells are not widely used for experiments in the context of structured surfaces. Cells of this type were able to grow on all hierarchically structured surfaces and no visual change in morphology was observed (Figure 4 column II).

A more interesting fact was discovered when observing the morphology of the mouse fibroblasts (Figure 4 column I). On the structured surfaces, the stress fibers in the fibroblast structure almost disappeared. Stress fiber formation is often associated with cultivation in a 2D environment. The substrates presented here form a so-called pseudo-3D model that is probably responsible for reducing the number of stress fibers in cells. As can be seen in Figure 4 column I, the occurrence of stress fibers was reduced, especially on surfaces with Meso/Micro and Macro/Micro structure. This finding proves the applicability of the prepared materials as the substrate capable of mimicking the in vivo tissue architecture.

**Figure 5:**
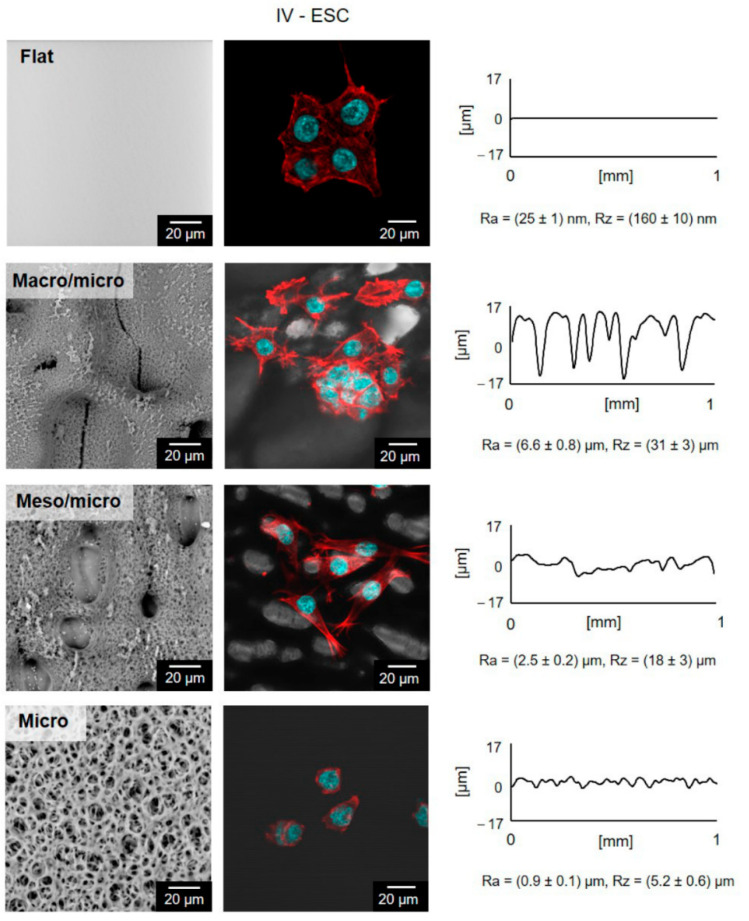
Embryonic stem cells (ESC) on the structured PS, column IV. Blue marked cell nuclei (Hoechst), red cytoskeleton (ActinRed). PS surfaces prepared by one or more stepwise time-sequenced phase separation (Macro/Micro and Meso/Micro) correspond to Figure 2. PS foam (Micro) prepared by phase inversion corresponds to Figure 3. Images from the SEM on the left, from the confocal microscope in the middle, and from the contact profilometer on the right.

The studied stem cells on “Micro PS”, as well as on flat surfaces, do not change the shape of their cytoskeleton, as seen in Figure 5. They are characterized by a compact shape with a dominant core (blue marked). While in contact with the hierarchically textured “Meso/Micro PS” or “Macro/Micro PS”, significant changes in the cytoskeleton distribution can be observed (marked in red). Regardless of the overall cell viability, the preferential orientation of the cytoskeleton can be monitored on the Meso/Micro surface in accordance with the orientation of the micropores. The cell nucleus is preferably located inside the pore, as in Figure 5. These changes may be related to stem cell differentiation as the shape of the cell is one of the markers of the developmental processes [52]. The influence of geometric properties on stem cell differentiation has been investigated in a number of studies [53,54,55,56]. For example, Kawano et al. [53] tested the effect of different pore sizes of PS material on human mesenchymal stem cells (hMSCs). MSCs are more advanced in development and, unlike ESCs, they are multipotent. However, they can also develop into several different types such as adipocytes, chondrocytes or osteoblasts. It was found that the larger the pore size, the more polygonal the shape of the cells. The work of Luo et al. [55] deals with the control of hMSC adipogenesis by changing the geometry of the substrate surface. They created various geometries on the substrate, such as pentagons, squares, trapezoids, and octagons. It has been established that geometric shapes play a role in the rate of adipogenic differentiation. Similarly, osteogenic differentiation of hMSC could be controlled by surface topography [54]. Furthermore, Ankam et al. [56] have tested the effect of topography on the differentiation of human ESCs into neural lines. Topography had no significant effect on cells cultured in a medium strongly promoting the maintenance of pluripotency. Nevertheless, in the case of using a neuro supporting medium, the influence of different geometry was recognized. Samples with an isotropic surface promoted the formation of glial cells, while anisotropic grids supported higher neuronal cell formation. Markert et al. on the contrary, used surface topography as a tool to retain the totipotence of mice ESCs without the need to use a non-differentiating medium [57].

Surface geometry clearly plays a role in influencing stem cell behavior. This is also pointed out in the results published here. Cells that had more space for differentiation and growth took on a polygonal shape, while cells growing on a surface with smaller pores took on a spherical shape similar to the undifferentiated state (Figure 4).

**Figure 6:**
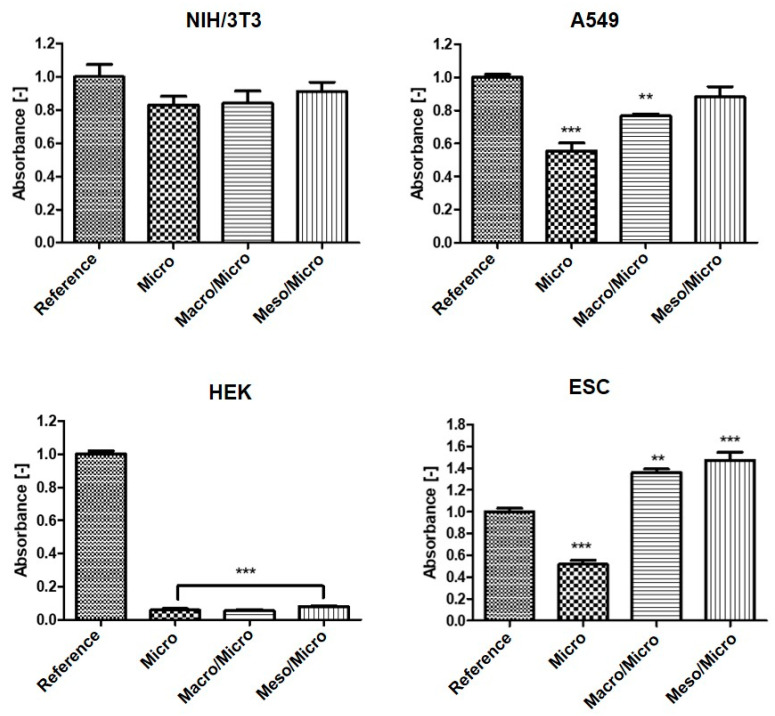
Quantification of initial growth/proliferation of different cell lines using MTT assay. NIH/3T3—fibroblasts, A549—human carcinoma cells, HEK—keratinocytes and ESC—embryonic stem cells. ANOVA with post hoc Tukey’s Multiple Comparison test was applied to determine any statistical differences between the samples; ** *p* < 0.01, *** *p* < 0.001.

In order to determine the line-specific response of cells to hierarchically structured surfaces, their quantification was also performed. The results are presented in the form of graphs, where the absorbance value correlates with the number of cells on the substrates. As shown in Figure 6, keratinocytes were able to proliferate only on the native untreated plastic. In the case of A549 lung tumor cells, the cellular ability to grow was significantly lower on the surface with Micro structure, the number of cells was also reduced on the surface with the Macro/Micro pores. In contrast, the structured substrates did not inhibit the growth of mouse fibroblasts, the measured absorbance values were almost identical to the absorbance on the native surfaces. The ESC cell line was the only cell line to show significantly better proliferation on both types of hierarchically structured surfaces, as shown in Figure 5. Thus, hierarchically structured surfaces have the potential to cultivate stem cells in particular, as they have exhibited good proliferation. Furthermore, the most significant visual change in morphology, which is usually associated with the cells differentiation, was observed, as shown in Figure 5 for Meso/Micro and Macro/Micro surface.

## 3. Materials and Methods

### 3.1. Materials and Reagents

Polystyrene (PS) Petri dishes with a diameter of 34 mm, sterilized by radiation, free from pyrogens and DNA/RNA for cell cultivation (TPP Techno Plastic Products AG, Trasadingen, Switzerland) were used as a substrate. Ultrapure water (18.2 MΩ.cm), tetrahydrofuran (THF, HPLC grade; Sigma-Aldrich Ltd., St. Louis, MO, USA), PS as polymer additive (TPP Techno Plastic Products AG) and 2-ethoxyethanol (ETH, p.a.; Sigma-Aldrich Ltd.) were used.

### 3.2. Solutions Preparation

Three types of solution mixtures for surface modification were used. Mixture 1 was THF with H_2_O in volume ratio of 5.6:4.4. Mixture 2 was THF with ETH in volume ratio of 1:2. Mixture 3 was prepared by dissolving 2.68 g PS in 10 mL THF for 3 h at 298 K. The 20 mL ETH was then added dropwise while Mixture 3 was constantly stirred. 

### 3.3. Preparation and Characterization of Hierarchically Structured Substrates

Surface preparation: For a deposition of solvent mixtures on PS dishes, a homemade spin coater was used [58]. The speed of rotation, dosed volume, number of doses and the delay of the individual doses were set as is indicated for the individual results. Solvent mixture was deposed from glass syringe with metal needle placed 30 mm above the center of a PS dish surface. After the last dose, the sample was left to rotate for an additional 120 s. All samples were modified at 298 K (substrate, solutions, surrounding atmosphere) and air humidity of 50%, as in our previous works [33,58]. For the purposes of this work the surface irregularities (pores) are referred to as Flat (under 0.1 µm), Micro (from 0.1 to 10 µm), Meso (from 10 to 50 µm) and Macro (over 50 µm). 

*Plasma treatment:* To increase the affinity of the cultivated cells to the structured PS, substrates were pre-treated by low temperature air plasma. The treatment was carried out in a Diener Femto plasma reactor with a capacitive coupling at a frequency of 13.56 MHz (Diener electronic GmbH + Co.KG, Ebhausen, Germany), a pressure of 1 mbar and an air-flow rate of 5 sccm (purity 99.999%). The forward power was set to 100 W and the reflected power was maintained at 10% with the help of the matching circuit during all experiments. The plasma processing time was 3 s. After treatment, the substrates were kept at a constant temperature of 298 K in a desiccator.

*Optical goniometry:* Static contact angles of water were measured and evaluated on the samples before and after plasma modification process by using the automated Drop Shape Analyzer—DSA30 and Advance software (KRÜSS GmbH, Hamburg, Germany). Subsequently, measurements were made on samples after one day interaction with culture medium to determine changes in contact angles. The measurement was performed as follows: 5 drops of ultrapure water with a volume of 3 μL were deposited on each measured surface at a room temperature of 298 K and 50% humidity. 

*Scanning electron microscopy:* Structured surfaces were observed by a scanning electron microscopy (SEM) model Phenom Pro (Phenom-World BV, Eindhoven, The Netherlands). Samples were analyzed at an acceleration voltage of 10 kV in backscattered electron mode, with the magnification from 1000× to 4000×. Measurements were carried out on samples without prior metallization, using a holder allowing the reduction of charges on polymeric materials.

*Atomic force microscopy:* The surface topography of flat and micro structured surfaces was characterized with an atomic force microscope (AFM), model NTEGRA-Prima (NT-MDT, Moscow, Russia). Measurements were performed at the scan rate from 0.5 to 1 Hz, with a resolution of 512 × 512 pixels in tapping mode at 298 K under the air atmosphere. A silicon-nitride probe NSG01 (AppNano, Mountain View, CA, USA) with resonant frequency of 150 kHz and a stiffness constant of 5.5 N·m^−1^ was used. The data from AFM were processed in the Gwyddion 2.55 software (Czech Metrology Institute, Jihlava, Czech Republic).

*Contact profilometry:* The changes in the surface topography and roughness for all samples were characterized by contact profilometer, model DektaXT (Bruker, Billerica, MA, USA). A tip with a radius of curvature of 12 µm and a pressure equivalent of 5 mg was used. The surface roughness values (Ra) and maximum height changes (Rz) were determined from 5 individual measurements according to the SME B46.1 standard.

*Optical profilometry:* 3D optical microscope Contour GT-K (Bruker) based on white light interferometry with use of 20× objective lenses was used to visualize surface topography. The resulting 2D topography maps were processed in the Gwyddion 2.55 software.

*Image processing and analysis:* Images from SEM were processed using the ImageJ software, version 1.5 (W. Rasband, National Institutes of Health, Bethesda, MD, USA), the scale bars were added.

### 3.4. Proliferation of Cells

All samples were treated by plasma before each cell proliferation test to increase the affinity of the cultivated cells to the structured PS. The cells were deposited on the surface within 2 h of plasma treatment to minimalize the phenomenon called aging—a gradual process of deterioration of the properties of the modified PS surface layer. Aging is explained in terms of the reorientation of functional groups incorporated at plasma treatment during time intervals [58,59,60,61]. To ensure evaporation of all residual solvents, the modified surfaces were placed into the hood with increased air flow for 24 h at 22 °C, then stored for 48 h in a desiccator and finally evacuated during plasma treatment. The subsequent control experiments have shown that this procedure was sufficient to remove any residual solvents that could affect the cultured cells. 

Mouse embryonic fibroblast: Mouse embryonic fibroblast cell line (ATCC CRL-1658 NIH/3T3; Marlboro, MA, USA) was used to test the proliferation of cells on the plasma treated surfaces. ATCC-formulated Dulbecco’s modified Eagle’s medium (Biosera, Nuaille, France) containing 10% calf serum (Biosera, Nuaille, France) and 100 U mL·mL^−1^ penicillin/streptomycin (PAA, Trasadingen, Switzerland) was used as the culture medium. Individual samples were sterilized by UV radiation for 30 min before the testing. An untreated PS Petri dish (TPP) was used as a reference sample (for each experiment).

A visualization of cell morphology and actin filament was undertaken. NIH/3T3 cells were seeded at an initial concentration of 1.105 cells·mL^−1^. Cultivation was carried out for 3 days, under suitable conditions: 37 °C in 5% CO_2_ in humidified air. After 3 days of proliferation, the cells were fixed and stained to visualize the DNA with Hoechst 33258 (Molecular Probes, Carlsbad, CA, USA) and actin fibers with ActinRed 555 (Life Technologies, Carlsbad, CA, USA). The fixed and fluorescently labeled cells on the surfaces were characterized by confocal laser scanning microscope, model FV3000 (Olympus, Tokyo, Japan). Microscopic objectives with magnification of 4× to 40× were used.

Human lung carcinoma cells: The effect of structured PS was also tested on human lung carcinoma cells (A549, CCL-185™, Marlboro, MA, USA). The culture medium and the process of proliferation were identical as for NIH/3T3 cells. 

Human epidermal keratinocytes: Keratinocyte Growth Medium 2 (Sigma-Aldrich; St. Louis, MO, USA) was used to cultivate primary epidermal human keratinocytes (HEK) on structured surfaces. The medium contained the following individual components necessary for keratinocytes culturing: 0.004 mL·mL^−1^ bovine pituitary extract, 0.125 ng·mL^−1^ epidermal growth factor, 5 μg·mL^−1^ insulin, 0.33 μg·mL^−1^ hydrocortisone, 0.39 μg mL^−1^ epinephrine, 10 μg·mL^−1^ transferrin and 0.06 mM CaCl_2_ (all from Sigma-Aldrich; USA).

The HEK were seeded at a density of 3000 cells·cm^−2^. Cultivation was carried out for 6 days, at 37 °C in 5% CO_2_ in humidified air. The culture medium was changed every 2 days. Cell morphology was visualized by DNA staining with Hoechst 33258 (Molecular Probes, Carlsbad, CA, USA) and actin fibers with ActinRed 555 (Life Technologies, Carlsbad, CA, USA) as mentioned above. 

Embryonic stem cells: The effect of structured PS surface on embryonic stem cell (ESCs) proliferation was performed using an ES R1 cell line [62]. The ESCs were propagated in an undifferentiated state by culturing on gelatinized tissue culture dishes in complete media. The gelatinization was performed using 0.1% porcine gelatin in water. The cultivation medium contained the following components: Dulbecco’s Modified Eagle’s Medium (DMEM), 15% fetal calf serum, 100 U·mL^−1^ penicillin, 0.1 mg·mL^−1^ streptomycin, 100 mM non-essential amino acids solution (all from Gibco-Invitrogen; Billings, MT, USA), 0.05 mM 2-mercaptoethanol (Sigma-Aldrich; USA) and 1000 U·mL^−1^ of leukemia inhibitory factor (LIF) (Sigma–Aldrich; USA). 

The ESCs were seeded at a density of 5000 cells·cm^−2^. Cultivation was carried out for 4 days, under suitable conditions: 37 °C in 5% CO_2_ in humidified air. The culture medium was changed after 2 days. After 4 days of proliferation, the cells were fixed and stained to visualize the DNA with Hoechst 33258 (Molecular Probes, Carlsbad, CA, USA) and actin fibers with ActinRed 555 (Life Technologies, USA) as mentioned above.

### 3.5. Quantification of Cells

To quantify the cell proliferation, the MTT assay was used (MTT cell proliferation assay kit (Duchefa Biochemie, Haarlem, The Netherlands). The absorbance was measured at 570 nm and the reference wavelength was fixed at 690 nm. The results are presented in the form of graphs, where the absorbance value correlates with the amount of cells on the structured surfaces. The tests were performed in quadruplicate. Statistical significance was determined by ANOVA with post hoc Tukey’s Multiple Comparison test; ** *p* < 0.01, *** *p* < 0.001.

## 4. Conclusions

The presented study showcases an easy and efficient technique for the preparation of structured polymeric surfaces applicable for the study of cell behavior in simulated three-dimensional biomimetic microenvironment.

For the treatment of polystyrene surfaces, a fast and reproducible procedure based on time-sequenced dosing of mixed solutions on a rotating surface was used. It has been found that with the help of specifically established one—or more—phase separation steps, hierarchically structured surfaces with irregularities in the range of units up to hundreds of micrometers can be prepared. If a mixture of solvents contains a polymer component, polymeric foams can be prepared. Their thickness is directly proportional to the number of deposited doses that can be prepared. The proposed procedures expand the possibilities of using phase separation and inverse processes for the preparation of porous systems and hierarchically textured layers without the use of additives. 

Four different cell lines were used to study the suitability of structured surfaces for biological applications. It was shown that structured surfaces inhibited keratinocyte growth. In the case of mouse fibroblasts, there was a reduction of stress fiber concentration on the meso and macro pore surfaces, which is associated with the influence of the so-called pseudo 3D structure. The embryonic stem cells react on the structured surfaces by changed morphology and thus even associated differentiation processes. 

It was found that without any external stimuli, the cells reacted on the structured surfaces by different behavior as presented by the changes in morphology. 

## Figures and Tables

**Table 1:** Wetting contact angles of compared surfaces with water.

PS Sample	Before Plasma Treatment [°]	After Plasma Treatment [°]	After Plasma Treatment and Application of the Cell Culture Media [°]
Flat	71 ± 1	*	*
Micro	104 ± 3	28 ± 7	23 ± 3
Meso/Micro	115 ± 8	27 ± 2	24 ± 4
Macro/Micro	121 ± 2	25 ± 2	28 ± 6

* The initial PS surface was not plasma treated. This surface was treated for cell cultivation by producer, TPP Techno Plastic Products AG.

## Data Availability

Not applicable.

## Supplementary Materials

The following supporting information can be downloaded at: https://www.mdpi.com/article/10.3390/ijms23052541/s1.
