# Supplementary material for: Hierarchically Structured Surfaces Prepared by Phase Separation: Tissue Mimicking Culture Substrate
Source: Int J Mol Sci. 2022 Feb 25;23(5):2541. doi: 10.3390/ijms23052541 (PMC8910751; doi:10.3390/ijms23052541)
Supplement: Supplementary file 1 [file ijms-23-02541-s001.zip › ijms-1596248-supplementary.pdf]

Supplementary information to article:

Hierarchically structured surfaces prepared by phase separation: Tissue mimicking culture substrate

Markéta Kadlečková [1,2], Kateřina Skopalová [2], Barbora Ptošková [1], Erik Wrzecionko [1,2], Eliška Daďová [2], Karolína Kocourková [1,2], Aleš Mráček [1,2], Lenka Musilová [1,2], Petr Smolka [1,2], Petr Humpolíček [1,2,], Antonín Minařík [1,2,*]*

[1] Faculty of Technology, Tomas Bata University in Zlin, 760 01 Zlin, Czech Republic

[2] Centre of Polymer Systems, Tomas Bata University in Zlin, 760 01 Zlin, Czech Republic

* Correspondence: E-mail: minarik@utb.cz, E-mail: humpolicek@utb.cz

Corresponding authors: E-mail: minarik@utb.cz, +420 576 03 5086, ORCID: 0000-0002-0055-675X, E-mail: humpolicek@utb.cz, +420 576 038 035, ORCID: 0000-0002-6837-6878

1. Roughness analysis of structured polystyrene surfaces

To visualize the surface topography of the polystyrene surfaces, the 3D optical microscope Contour GT-K (Bruker) was used. Resulting topography was analyzed in the Gwyddion 2.55 software (D. Nečas, P. Klapetek, Czech Metrology Institute, Czech Republic). Profile (2D) roughness parameters Ra (arithmetical mean roughness) and Rz (mean roughness) were obtained by the contact profilometer DektaXT (Bruker) by measuring roughness profiles of

each sample. Areal roughness parameters Sa (area arithmetical mean height) were obtained by the 3D optical microscope with software Vision64 (Bruker).

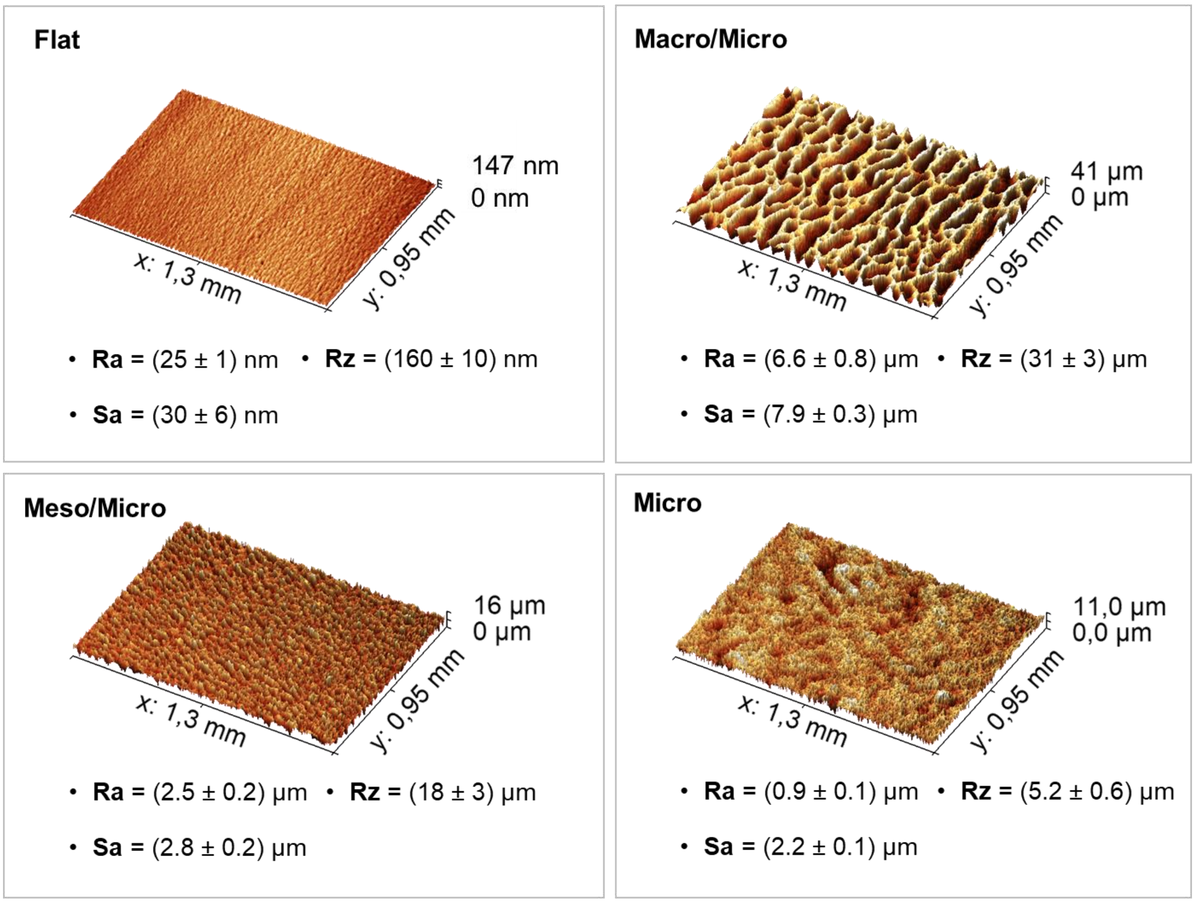

Figure S1 *– 3D images of polystyrene surfaces and resulting roughness parameters for each sample.*

2. Pores analysis of structured polystyrene surfaces

For pore imaging of prepared surfaces, the 3D optical profilometer (for Macro/Micro and Meso/Micro structured PS surfaces) and scanning electron microscopy (SEM) (for Micro structured PS surface) were used. To analyze the data from the 3D optical microscope, the Gwyddion v. 2.55 software (D. Nečas, P. Klapetek, Czech Metrology Institute, Czech Republic) and ImageJ v.1.5 software (W. Rasband, National Institutes of Health, United States) were used.

- First, the cross sections of 3D topography obtained by 3D optical microscopy were executed using Gwyddion software for Macro/Micro and Meso/Micro structured PS surfaces. For Macro/Micro PS surface the cross section was taken in the height of 15 µm and for meso/micro in the height 5 µm. The analyzed area for **Macro/Micro** and **Meso/Micro** pores evaluation was **700×700 µm**.

The Micro surface was analyzed from the photos taken by a SEM, since the pores of this surface are very small compared to the macro and the analysis from the 3D topography would be inaccurate. The analyzed area for **Micro** pores evaluation was **70×70 µm**.

- To get the pores outlined and numbered (Figure 2 – B, D, F) the function “Analyze particles” in software ImageJ v.1.2 was used. Then the resulting data were exported in the form of histogram (Figure 2).

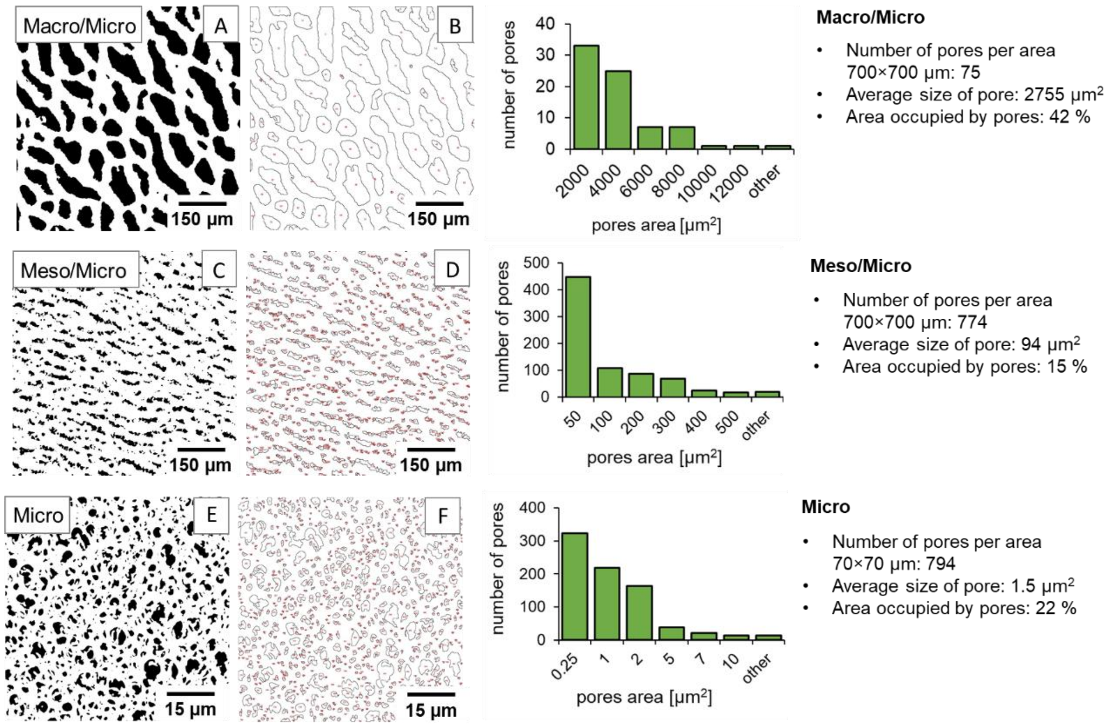

Figure S2 *- 2D images of cross sections or SEM image with highlighted and outlined pores for A, B - Macro/Micro; C, D – Meso/Micro and E, F – Micro structured PS surfaces.*
